# Characterisation of tissue factor-bearing extracellular vesicles with AFM: comparison of air-tapping-mode AFM and liquid Peak Force AFM

**DOI:** 10.3402/jev.v2i0.21045

**Published:** 2013-08-27

**Authors:** Julie Hardij, Francesca Cecchet, Alexandre Berquand, Damien Gheldof, Christian Chatelain, François Mullier, Bernard Chatelain, Jean-Michel Dogné

**Affiliations:** 1 Department of Pharmacy, NARILIS, Namur Thrombosis and Hemostasis Center (NTHC), University of Namur, Namur, Belgium; 2 Research Centre in Physics of Matter and Radiation, NARILIS, University of Namur, Namur, Belgium; 3 Bruker Nano, Heidelberg, Germany; 4 Hematology Laboratory, NARILIS, Namur Thrombosis and Hemostasis Center (NTHC), CHU Mont-Godinne, Université Catholique De Louvain, Louvain-La-Neuve, Belgium

**Keywords:** extracellular vesicles, tissue factor, atomic force microscopy, coagulation, biomarker

## Abstract

**Introduction:**

Extracellular vesicles (EVs) are shed from cells and carry markers of the parent cells. Vesicles derived from cancer cells reach the bloodstream and locally influence important physiological processes. It has been previously shown that procoagulant vesicles are circulating in patients’ fluids. These EVs are therefore considered as promising biomarkers for the thrombotic risk. Because of their small size, classical methods such as flow cytometry suffer from limitation for their characterisation. Atomic force microscopy (AFM) has been proposed as a promising complementary method for the characterisation of EVs.

**Objectives:**

The objectives of this study are: (a) to develop and validate AFM with specific antibodies (anti-TF) and (b) to compare air and liquid modes for EVs’ size and number determination as potential biomarkers of the prothrombotic risk.

**Methods:**

AFM multimode nanoscope III was used for air tapping mode (TM). AFM catalyst was used for liquid Peak Force Tapping (PFT) mode. Vesicles are generated according to Davila et al.'s protocol. Substrates are coated with various concentrations of antibodies, thanks to ethanolamine and glutaraldehyde.

**Results:**

Vesicles were immobilised on antibody-coated surfaces to select tissue factor (TF)-positive vesicles. The size range of vesicles observed in liquid PFT mode is 6–10 times higher than in air mode. This corresponds to the data found in the literature.

**Conclusion:**

We recommend liquid PFT mode to analyse vesicles on 5 µg/ml antibody-coated substrates.

Microvesicles (MVs) are small entities defined by a size inferior to 1 µm. They are vesicles budding from the membrane after an activating or apoptotic stimulus that increases the calcium release from the endoplasmic reticulum (1,2). This increase in intracytoplasmic calcium concentration weakens the membrane stability. The floppase and the scramblase enzymes are activated and the flippase is inhibited which leads to the loss of the cell membrane asymmetry. Exosomes are small vesicles (<100 nm) originating from multivesicular bodies that are released after their fusion with the plasma membrane. Exosomes and MVs are part of the whole extracellular vesicle (EV) population and may be composed of lipids, mRNA and proteins from the parent cell (3,4). Every cell is thought to be capable of generating EVs. In healthy individuals, EVs are found circulating in the plasma. They originate from endothelial cells, monocytes, red blood cells and platelets. Microvesicles attract more and more consideration because their concentration is found elevated in a series of diseases (5). Indeed, patients with cardiovascular disease or cancer have an elevated number of circulating particles in their plasma compared to healthy individuals. EVs are implicated in important mechanisms, such as inflammation, angiogenesis, metastasis (6) or thrombosis (7). Our hypothesis is that EVs are implicated in thrombosis due to their ability to carry key factors capable of ectopically initiating the coagulation. Among them, the tissue factor (TF) is a potent activator of the coagulation cascade (8,9). It is a 47-kDa protein also known as coagulation factor III. It binds and activates the serine protease factor VII and triggers the extrinsic pathway that leads to thrombin generation and fibrin clot formation.

Therefore, detecting the level of TF-positive EVs may lead to an adapted prophylaxis for cancer patients (10,11).

The count and size distribution of these particles are subject to controversy. The current method routinely used to characterise EVs is flow cytometry (FCM) (12). However, this method is limited by its sensibility that only allows the detection of EVs>300 nm and size calibration issues (13). Other more specific methods include DLS (dynamic light scattering), SEM (scanning electron microscopy) or TEM (transmission electron microscopy) (14,15). Electron microscopy techniques are suitable for EVs visualisation but the pre-analytical steps associated with these techniques may lead to alteration of the EVs. DLS provides a size distribution but no biological information (i.e. protein surface characterisation). To overcome the limitations of the above techniques, the use of atomic force microscopy (AFM) was proposed as a more accurate technique to measure the EVs (16). Furthermore, the use of antibody-coated micas allows for the selection of a particular population of EVs giving information on present surface antigens in a physiological environment (17). These main advantages make AFM a very promising tool to precisely characterise EVs, ranging from the nanometre to the micrometre scale (18,19).

The AFM can be used in different acquisition modes: air or liquid. The air condition is more stable but implicates a sample-drying process. Conversely, the liquid mode may reflect a more real state of the vesicles. Yuana et al. measured platelet-derived vesicles in healthy donors and cancer patients with AFM in liquid mode. No size or count differences were detected between both individual conditions.

The objectives of this study are to: (a) develop and validate AFM with specific antibodies (anti-TF); and (b) compare air and liquid modes for EVs’ size and number determination as potential biomarkers of the prothrombotic risk.

## Materials and methods

### Cell culture

The breast cancer cell line MDA-MB-231 (ATCC ref. number HTB-26) was chosen for their capacity to release large amounts of EVs (20). This cell line is also known to express the TF protein on its surface. Culture media is RPMI 1640 (BE12-702F) completed with 10% FBS (DE14-801F) and 1.5 g/l sodium bicarbonate (BE17-613E, Lonza). They were maintained in 5% CO_2_. Washes are made with PBS (BE17-516F, Lonza). Cells were detached from their substrate by using a trypsin-EDTA solution (BE02-007E, Lonza), which was used at 0.007 g/ml concentration. Cells were stocked in liquid nitrogen in a 10% DMSO (dimethylsulfoxide) solution (D8418-50 ML, Sigma Aldrich) and 10% FBS.

### In vitro generation of EVs

A modified version of the protocol reported by Davila et al. (20) was used. Cells were cultured until confluence and then trypsined and counted. They were adjusted to the desired density of 12×10^6^/ml in PBS and incubated for 45 minutes at 37°C without any stirring. The PBS solution containing the cells and vesicles was centrifuged at 200 g for 5 minutes to pellet the cells. The PBS supernatant containing the EVs was used directly for staining with antibodies. PBS alone was used as a negative control.

#### Platelet-free plasma preparation

Platelet-free plasma was obtained as previously described (21). The blood is submitted to a first centrifugation at 2,500 g for 15 minutes at room temperature (Heraeus Multifuge 1S-R, Sysmex Benelux, Etten-Leur, The Netherlands) within 1 hour after sampling according to Mullier et al. (22). A light break is applied. The platelet-poor plasma was collected and transferred into a polypropylene haemolysis tube with a micropipette. Aspiration was stopped 1 cm above the buffy-coat while avoiding the buffy-coat. Platelet-poor plasma was centrifuged a second time at 2,500 g for 15 minutes at room temperature. The platelet-free plasma was collected into a fresh tube using a micropipette, while leaving approximately 100 µl at the bottom of the tube.

### Methodology for antibody coating

The surface functionalisation protocol is based on the selective recognition between EVs and a complementary surface-immobilised antibody (23).

**Fig. 1:**
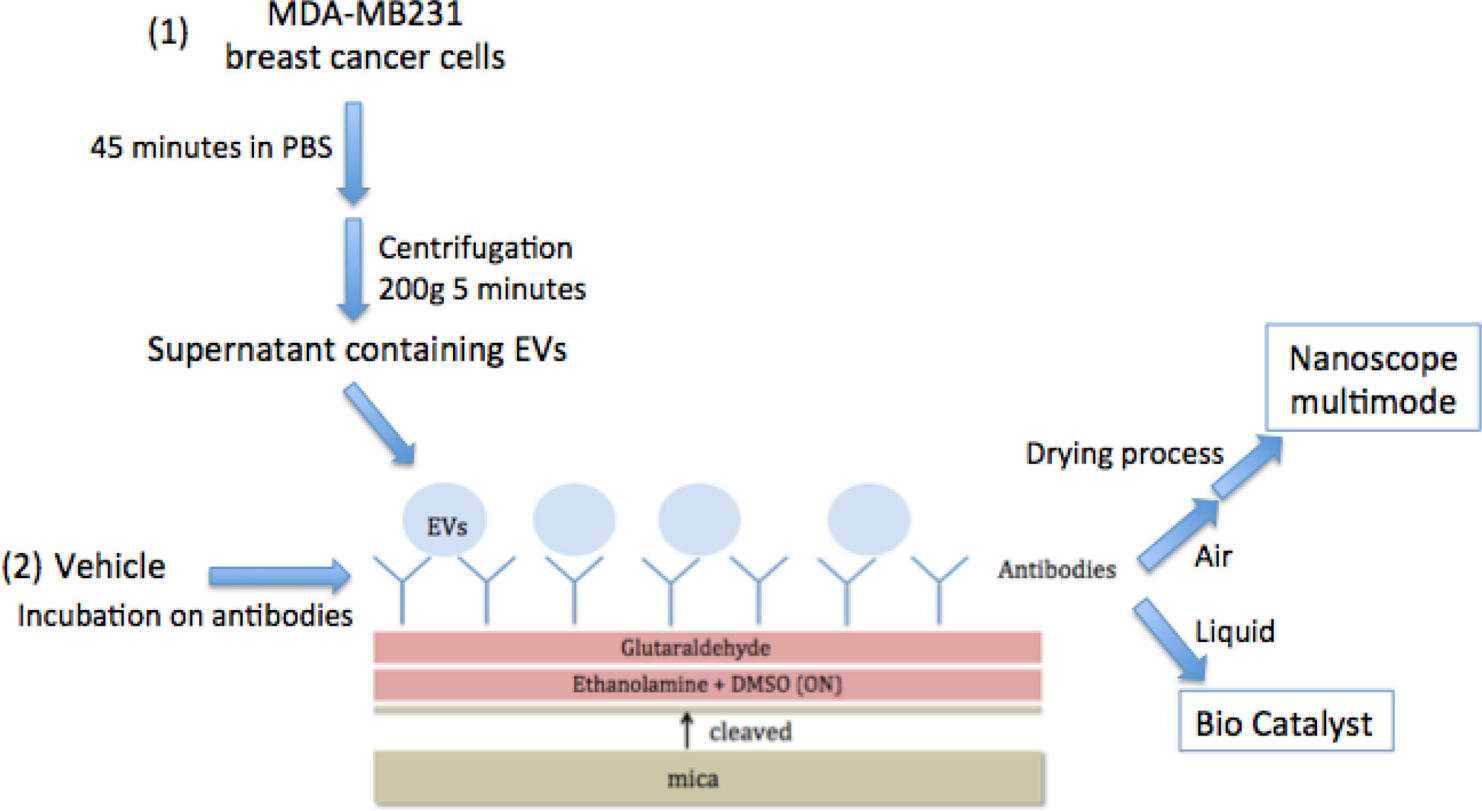
Schematic representation of mica functionalization process for AFM observation in 2 modes. Mica is first cleaved; the surface is incubated with ethanolamine and the glutaraldehyde after water and PBS washes. Antibodies are grafted for 1 hour on mica's surface and EVs (1) or controls (2) are incubated on the functionalised surface. These micas are ready for liquid observation or are dried for air observation.

Mica sheets were used as the solid support and then functionalised as follows (see Fig. 1). Freshly cleaved muscovite mica sheets (V-1 Grade, Electron Microscopy Sciences, Hatfield, PA, USA) with a diameter of 9.5 mm were incubated overnight at room temperature in a 55% (w/v) solution of ethanolamine (≥99.5%, Sigma Aldrich) in distilled dimethylsulfoxide (DMSO). This solution was first heated at 70°C to allow ethanolamine dissolving in DMSO. The above amine-functionalised mica sheets where then abundantly rinsed in DMSO and in absolute ethanol (≥99.8%, Fluka), dried under a stream of nitrogen, and then immediately activated for further grafting of the antibodies. Amine activation was carried out in a 7% (v/v) solution of glutaraldehyde in a phosphate buffer solution (0.1 M) for 30–60 minutes at room temperature. The mica sheets were then rinsed in phosphate buffer solution and then in Milli-Q water (18.2 MΩ·cm), and immediately covered by 25 µl of CD142 antibody solution (clone TF9-10H10 IgG1, Calbiochem^®^ ref 612161) for 30 minutes at room temperature. The concentration was 10 µg/ml for all experiments except when stated otherwise. CD142 is the marker of the TF protein. Excess of antibody was removed by rinsing with phosphate buffer and Milli-Q water.

Anti-CD41 (platelet marker) antibody was used as a negative control for EV coating (Millipore, ref: CBL130). Recombinant TF (American diagnostic ref 4500PC) was also used to assess that no EV is attached to antibody-saturated surfaces.

#### Vesicle attachment

A drop of 25 µL of EV suspension derived from MDA-MB231 or the negative control was applied to the antibody-coated mica surfaces at room temperature for 1 hour to allow EVs to recognise the antibody. The surfaces were then rinsed with buffer phosphate (1) and observed in liquid mode or (2) rinsed with Milli-Q water and dried before air measures.

## EV AFM analysis and counting

Micas prepared as described above were analysed according to 2 modes. For each image, a flatten process was applied. To count the vesicles, only 1×1 µm^2^ images are considered. This size is the only one that provides sensitivity.

### 

#### Air-mode AFM analysis

AFM images were recorded in air, in intermittent-contact mode (IC-AFM) with a Nanoscope III from Veeco Instruments (Santa Barbara, CA, USA). The cantilevers (Tap300Al-G from Budget Sensors) were silicon cantilevers with a resonance frequency around 300 kHz and a typical spring constant of around 40 N/m, and an integrated silicon tip with a nominal apex radius of curvature <10 nm. So-called soft-tapping conditions were used, that is the ratio between the set-point amplitude and the free amplitude of the cantilever vibration was always kept above 0.8. Images were recorded at 10×10 µm^2^, 3×3 µm^2^, and 1×1 µm^2^ sizes with 512×512 lines per image. At least 3 images of each enlargement were acquired and a sum of 1,000 vesicles was counted from several 1×1 µm^2^ for statistics. The topographical analysis of AFM images—that is grain size and density—was carried out by using the Nanoscope Analysis and SPIP software version 4.1.8.0. The resulting size estimation was expressed as mean±standard deviation (SD). According to Yuana et al., the EV concentration (EVs L^−1^) was determined from the relation (10^6^/10^3^×25)×(70.8×10^6^)×*N*_AFM_, where 10^6^ is the conversion factor from µl to l. One microlitre is the original volume of EVs containing PBS, 25 (µl) is the volume of the EV suspension applied to the surface, 70.8×10^6^ (µm^2^) is the area of the mica sheet, 1 µm^2^) is the size of the AFM image, and *N*_AFM_ is the number of TF-positive EVs per image. This formula is based on the hypothesis that all EVs adsorb onto the anti-CD142 antibody surface.

#### Liquid-mode AFM analysis

AFM images recorded in liquid were performed on the Bioscope Catalyst (Bruker, Billerica, USA) with MLCT probes (Bruker, Billerica, USA) in PeakForce Tapping mode (TM). Peak Force Tapping (PFT), also called Peak Force QNM (PFQNM) in quantitative mode, is an oscillating mode developed in 2009 to characterise a wide range of samples. Unlike in TM, the z-piezo is oscillated far below (1–2 kHz) the resonance frequency. While the cantilever is oscillated, a force curve is performed each time the tip interacts with the surface. Similar to TM, the interaction time is very short, thus lateral and shear forces are negligible. Unlike in TM where the feedback loop keeps the vibration amplitude constant, PFT controls the maximum loading force (so-called peak force) applied onto the sample. The key parameters to optimise the tracking are the peak force setpoint and the gains. That differs from TM where the applied force is controlled by the ratio between the amplitude setpoint and the free amplitude of oscillation. In PFT, the applied forces can be minimised to a few tens of pico Newton, which is below what is usually achievable with TM.

Each tip was calibrated prior to the experiments so that all displayed channels were directly quantitative. The calibration was performed as follows: (a) approach on a non-compliant part of the sample (mica), capture of a representative force curve and update of the deflection sensitivity in the linear part of the curve; (b) withdrawal and thermal tuning of the cantilever to calculate the spring constant; and (c) estimation of the tip radius via a tip-check sample. This step is mandatory to have accurate Young's modulus. The average applied for was in the order of 150 pN. The image is generated thanks to a force/distance curve captured on each image pixel with 256×256 lines per image. Several signals can be extracted in real time and simultaneously: the adhesion (vertical distance between the baseline and the lowest point of the retraction curve), the peak force (vertical distance between the baseline and the turn-away point), the deformation (horizontal distance between the turn-away point and the contact point), Young's modulus (by extrapolating the linear portion of the retraction curve; 2 fit models can be used: DMT, for poorly compliant samples, and Sneddon, for more compliant samples) and the dissipation (by integrating the area between the approach and the retraction curves). The main benefits of using PFT versus other techniques such as force spectroscopy (which is extremely slow) or TM (where the phase signal is a contribution of several factors that cannot be extracted quantitatively) can be summarised as follows:

- The applied force can be minimised to a few pico Newtons.
- Parameter adjustment is much easier (no tuning is required since the system always operates at a fixed frequency).
- All signals can be extracted individually and are directly quantitative.
- As a force curve is captured for each image pixel, the resolution will be the same as on the height image on all of the channels.

The statistics was performed via the Bearing Analysis function of Nanoscope Analysis (Bruker, Billerica, USA).

## Results

### Assessment of the coating protocol

**Fig. 2:**
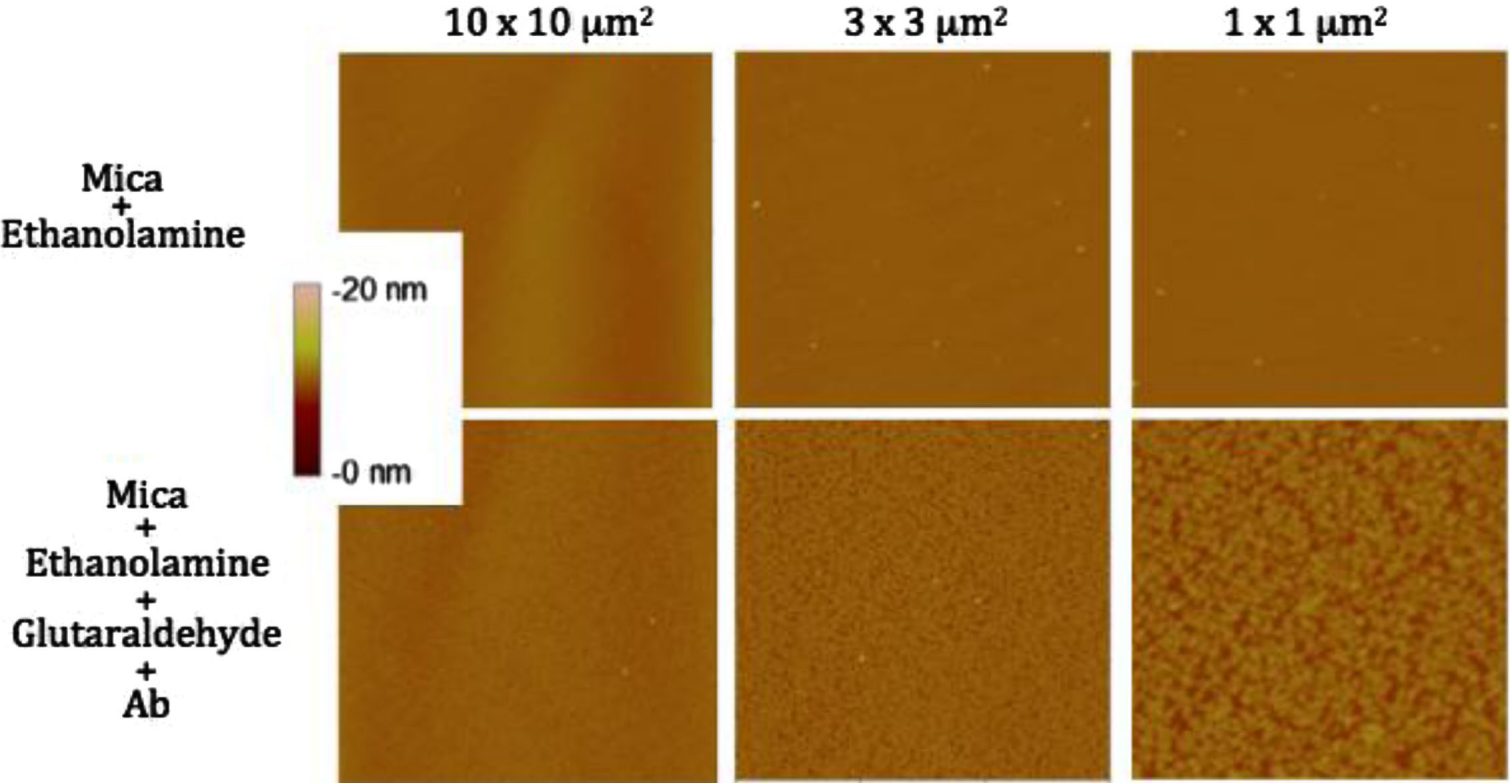
Images taken in air mode of the mica functionalisation steps. First line represents the mica after ethanolamine incubation; second line shows the mica after antibodies grafting. Three image sizes were considered: 10×10 µm^2^, 3×3 µm^2^ and 1×1 µm^2^. The colorimetric scale indicates the *Z* dimension.

**Fig. 3:**
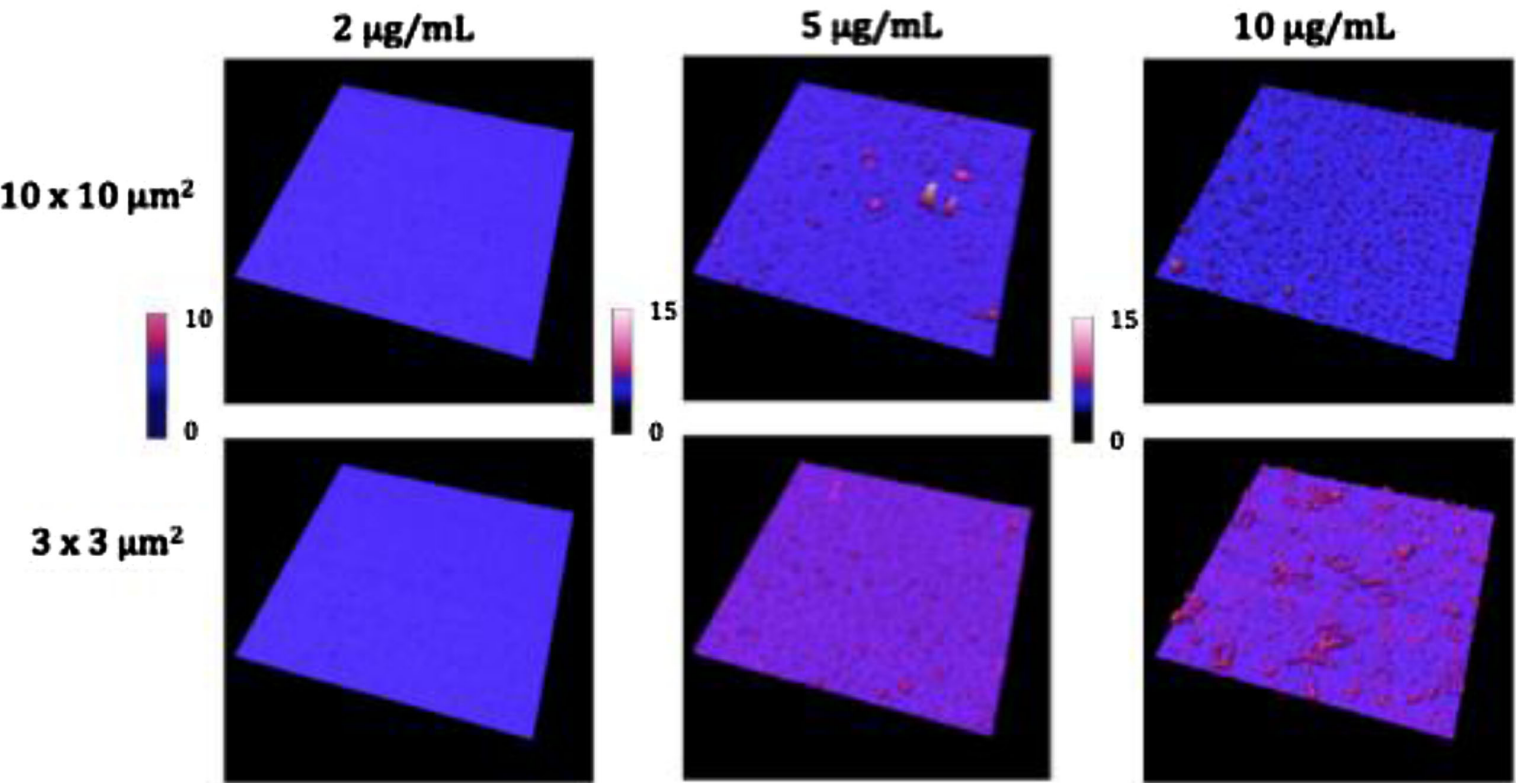
Images taken in liquid mode of the coating of 3 different concentrations of HTF1 (anti-TF) antibodies on mica surfaces. Column 1 is 2 µg/ml, column 2 is 5 µg/ml and column 3 is 10 µg/ml. Images were taken in 2 size windows: line 1 is 10×10 µm^2^ and line 2 is 3×3 µm^2^. *Z* scales are presented in nanometres below the images.

Images from air and liquid modes show the cleanliness of the surface after the steps of the functionalisation protocol (Figs. 2 and 3). Indeed, no vesicle-like artefact was observed right after the mica cleavage, neither after the ethanolamine incubation nor on antibodies’ layer. The antibody molecular diameter was about 20±4 nm (air) and 11.2±6.8 nm (liquid), this being the limiting size below which EVs cannot be distinguished from antibodies. The number of CD142 antibodies on the surface was estimated to approximately 2,200/µm^2^ in air mode. In liquid mode, 3 dilutions were prepared and incubated on the reactive surface (Fig. 3). Table 1 indicates that a 5 µg/ml concentration allows for the best surface covering. Images recorded on different areas of the sample indicated a highly homogeneous distribution of CD142 antibodies on the surface in both modes. For further measurements, a 10 µg/ml antibody concentration was applied in order to compare the same conditions.

**Table 1:** Coverage and height statistics of antibodies grafting on mica surfaces for 3 dilutions based on liquid images (Fig. 4).

	2 µg/ml antibodies	5 µg/ml antibodies	10 µg/ml antibodies
% of surface coverage	1.5±0.8	69.2±12.3	43±12.4
Average height (nm)	1.7±0.4	5.8±4.3	11.2±6.8

### Assessment of the specific coating of the EVs

MDA-MB231 breast cancer cell-derived EVs were incubated on the non-specific anti-CD41-(platelet marker) coated surfaces and no vesicle was observed on antibodies’ layer. This antibody was chosen in order to prove that when analysing plasma EVs cancer cell-derived EVs would not be mistaken with platelet-derived EVs.

HTF1-coated surfaces were also incubated with recombinant TF proteins (10 µg/ml) for 1 hour at room temperature and then rinsed. Twenty five microlitres of MDA-MB231 breast cancer cell-derived EVs were then incubated and no EV was retained on antibodies (data not shown).

### EV sizing and counting with both microscopes

**Fig. 4:**
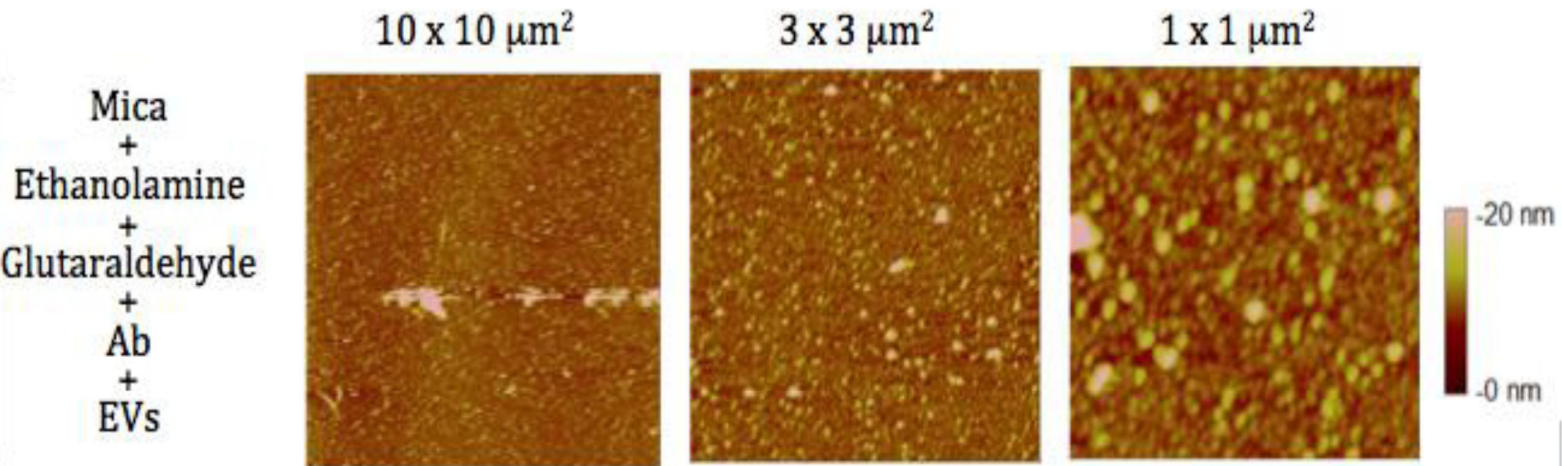
Images taken in air mode of the micas after the incubation of breast cancer cells-derived extracellular vesicles (EVs) on the anti TF-antibodies. Three image sizes: 10×10 µm^2^, 3×3 µm^2^ and 1×1 µm^2^. The colorimetric scale indicates the *Z* dimension.

**Fig. 5:**
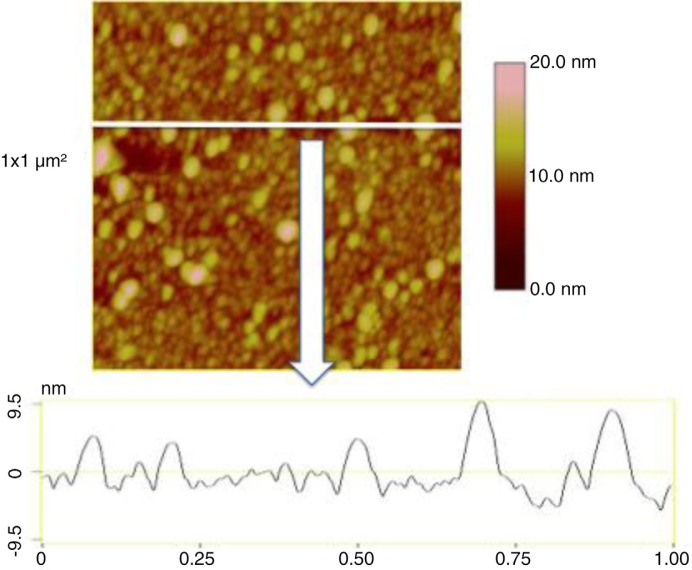
Image of a cross-section taken in air mode of the breast cancer cell-derived extracellular vesicles (EVs) on the anti TF-antibodies. Image size: 1×1 µm^2^. The colorimetric scale indicates the *Z* dimension.

In air mode, EVs were incubated on antibodies’ layer and gently rinsed and dried before AFM observation (see process in Fig. 1). The mean diameter of EVs released by MDA-MB-231 cells, as estimated from 1,200 EVs, was 38±9 nm (range: >20–60 nm) (Fig. 4). In the same AFM images, information of the EVs *Z* dimension devised from the graduated colour scale. The TF-EV concentration produced by 12×10^6^ MDA-MB231/ml was between 170×10^9^ and 170×10^10^ MVs l^−1^. In Fig. 5, a cross-section is provided to better represent the 3D profile of vesicles.

The EV suspension derived from MDA-MB231 showed 75,000 particles >20 nm on the same surface. One should notice that the PBS vehicle contained no particle.

**Fig. 6:**
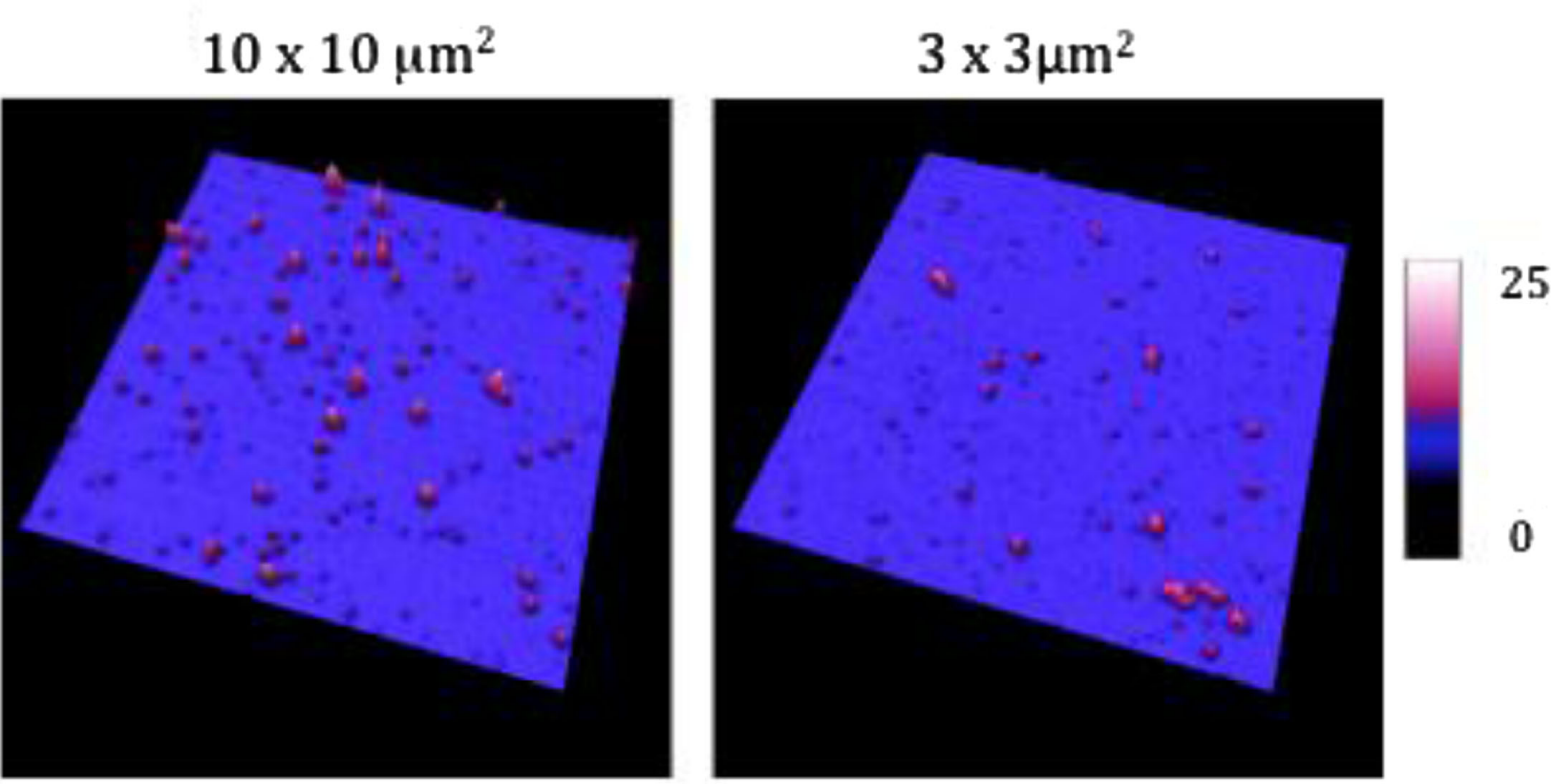
Images taken in liquid mode of breast cancer cell-derived EVs coated on anti-TF antibodies in 2 size windows: 10×10 µm^2^ and 3×3 µm^2^. The *Z* scale represents the sample height in nanometre.

**Fig. 7:**
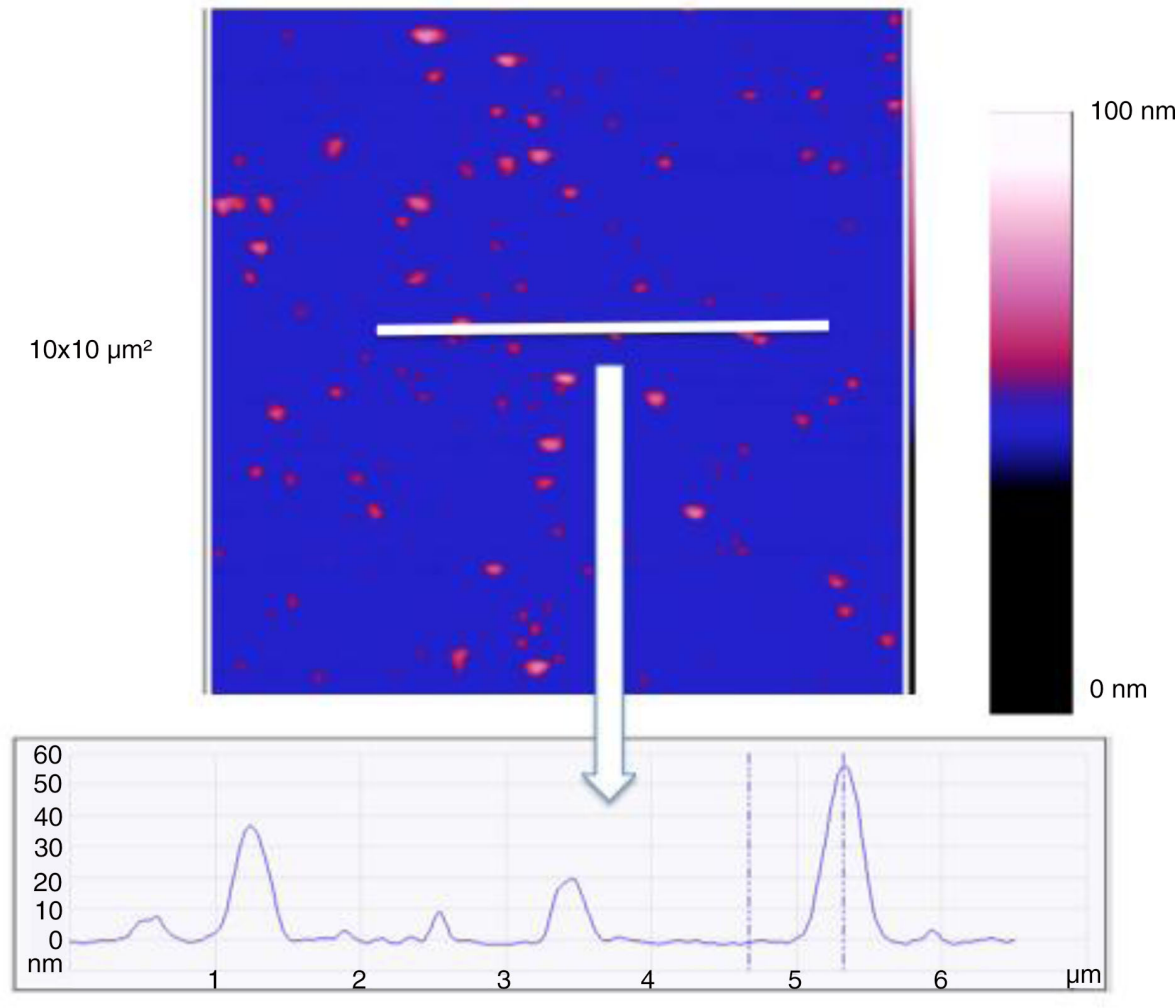
Image of a cross-section taken in liquid mode of the breast cancer cell-derived extracellular vesicles (EVs) on the anti-TF antibodies. Image size: 3×3 µm^2^. The colorimetric scale indicates the *Z* dimension.

In liquid-mode EVs from MDA-MB-231 breast cancer cells are detected on the anti-TF antibodies’ layer (Fig. 6). The size analysis of 256 vesicles revealed an average diameter of 219 nm of (range: 110–651 nm). Figure 7 shows a cross-section to represent the height of the vesicles in this mode.

### Healthy donor plasma-derived EV measurement

**Fig. 8:**
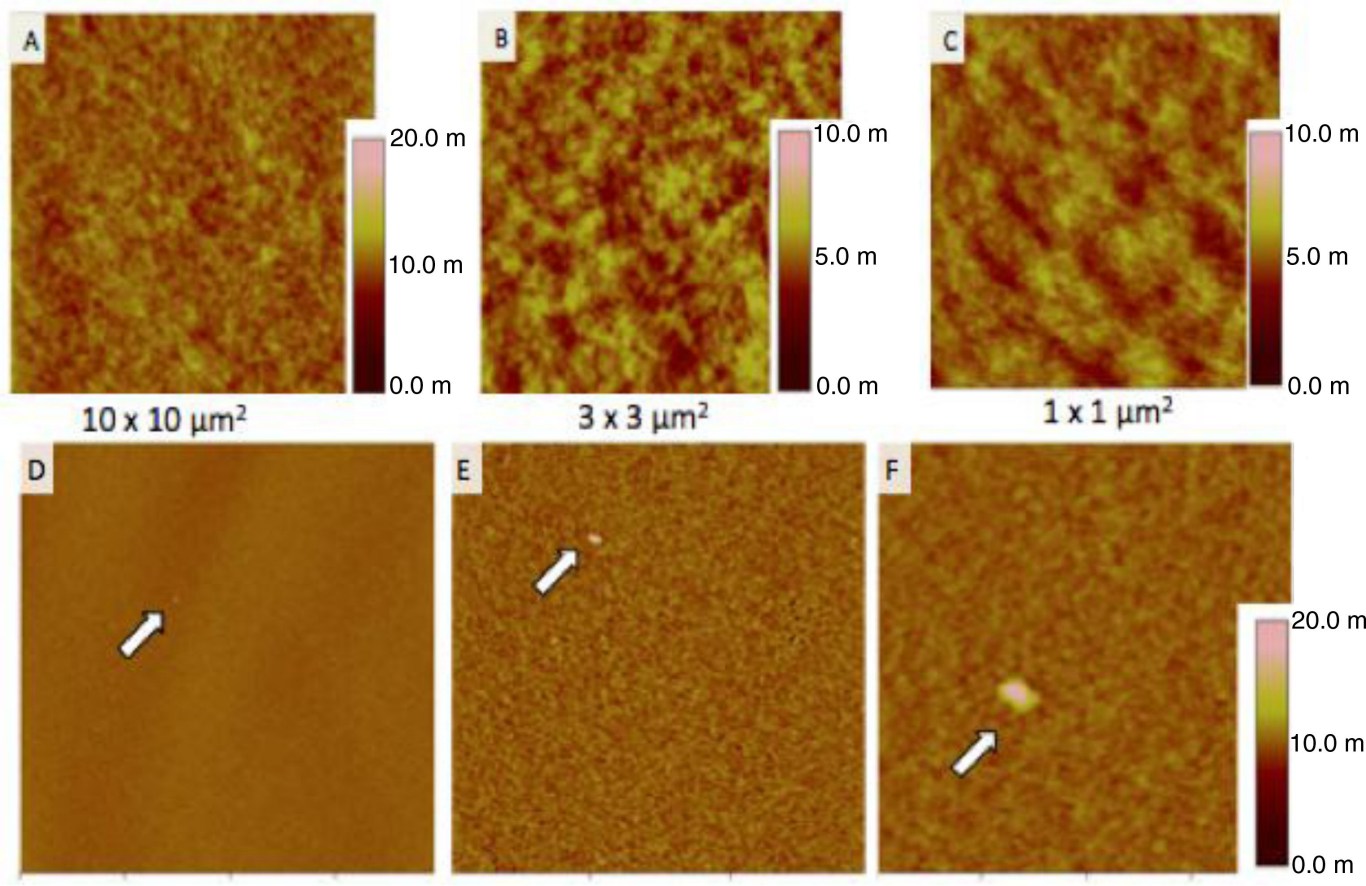
Images taken in air mode of healthy donor plasma incubated on IgG1 antibodies in 3 different size windows (A, B, C) and on anti-TF antibodies in 3 different size windows (D, E, F). Arrows show vesicles. The colorimetric scale indicates the *Z* dimension.

A preliminary test was also performed with one healthy donor plasma in air conditions. In physiological condition, vesicles are shed in the peripheral blood. Figure 8 shows images in 3 different size windows of plasma vesicles incubated on the negative control IgG1 (A, B, C). Also, plasma vesicles incubated on TF-coated mica (D, E, F). On dozen of large images, only 4 vesicles were observed with a size range of 60–100 nm. PBS was used as a negative control.

## Discussion

In order to fully characterise EVs, a combination of techniques is needed. A highly sensitive technique that rapidly and simultaneously gives a size distribution and an expression profile is required.

The AFM is able to image particles in 3 dimensions (height, width and length) with resolution and sensitivity down to the sub-nanometre range. By using mica sheets coated with specific antibodies, information on biochemical composition of EVs can be obtained. Furthermore, by using antibodies directed against a specific cell marker, the cell origin can be assessed.

In this study, we compared 2 conditions of AFM image acquisition: in air with TM and in liquid with PFT mode. The most relevant antibody concentration was first investigated for air and liquid modes. Ten microgram per milliliter is the best concentration for air measures and 5 µg/ml is recommended for liquid measurements. Beyond such concentrations, the antibodies aggregate. We specifically recommend the liquid mode for its conditions closer to physiological conditions.

The size of vesicles on images performed in air conditions may be an underestimation of the real size due to the drying process. Indeed, the liquid may evaporate under the airflow as no fixation is previously performed. On the contrary, liquid measurements definitely reflect the native size of the vesicles. Indeed, we highlighted a difference in the mean size of almost 6 times between air and liquid measurement. The ability of AFM to image biological samples in aqueous fluids enables the preservation of sample properties in their physiological state and allows for the determination of the vesicles size distribution with high precision. This technique was previously proposed by Yuana in 2010 (16) for vesicles size or number assessment but the antibody was less concentrated on the surface, so less vesicles were detected. We did not choose her this protocol because with it, we did not achieve a clean surface before the vesicle incubation (Supplementary Figure).

Air images of healthy donor plasma-derived vesicles were recorded. These measurements remain to be performed and validated in liquid mode.

This work is the first study that described the use of AFM that could be potentially used to detect TF-positive EVs in patients. Based on this technology, we showed that TF-EVs released by MDA-MB-231 were sized from 20 to 60 nm in air conditions and from 110 to 652 nm in liquid. This difference in sizes may be due to shrinkage of the vesicles during the drying process prior to air measurements.

Although interesting, this technique also suffers from some limitations. Similar to TEM, AFM count is based on the hypothesis that all particles are present in the image by adsorbing onto the surface. This assessment is reasonable and constant between each measure. This postulate can be controversial but might well reflect the whole vesicle population. A validation process remains to be performed in order to standardise protocols. We think that this technique could be used in routine clinical laboratoris to provide vesicle profile related to a particular pathology. Characterising the vesicles with regard to membrane expression such as the activated TF could constitute a biomarker for thrombotic risk for example. Furthermore, this assessment can be applied to all pathologies associated with an increased number of circulating vesicles. The standardisation should be performed with a standard solution of biological vesicles. Both modes can be used for the coating check but we recommend the liquid mode for vesicles analysis, as it is closer of physiological conditions.

Practically, the manipulation remains time-consuming but may be reduced by the use of pre-coated mica.

In conclusion, we showed that the use of AFM is very sensitive in the analysis of EVs. The results are promising and suggest that the method could be used in clinical laboratoris to study the prothrombotic profile of patients suffering from pathologies associated with an elevated number of vesicles.
